# Comparison between gastric and esophageal classification system among adenocarcinomas of esophagogastric junction according to AJCC 8th edition: a retrospective observational study from two high-volume institutions in China

**DOI:** 10.1007/s10120-018-0890-2

**Published:** 2018-11-02

**Authors:** Kai Liu, Fan Feng, Xin-zu Chen, Xin-yi Zhou, Jing-yu Zhang, Xiao-long Chen, Wei-han Zhang, Kun Yang, Bo Zhang, Hong-wei Zhang, Zong-guang Zhou, Jian-kun Hu

**Affiliations:** 1 Department of Gastrointestinal Surgery and Laboratory of Gastric Cancer, State Key Laboratory of Biotherapy, West China Hospital, Sichuan University and Collaborative Innovation Center for Biotherapy, No. 37 Guo Xue Xiang Street, Chengdu, 610041 Sichuan China; 2 Division of Digestive Surgery, Xijing Hospital of Digestive Diseases, Fourth Military Medical University, 127 West Changle Road, Xi’an, 710032 Shanxi China; 3 West China School of Medicine, Sichuan University, Chengdu, Sichuan China; 4 Department of Gastrointestinal Surgery and Laboratory of Digestive Surgery, Institute of Digestive Surgery and State Key Laboratory of Biotherapy and Cancer Center, West China Hospital, Sichuan University and Collaborative Innovation Center for Biotherapy, No. 37 Guo Xue Xiang Street, Chengdu, 610041 Sichuan China

**Keywords:** 8th TNM classification, Esophagogastric junction adenocarcinoma, Esophageal cancer, Gastric cancer

## Abstract

**Background:**

The new 8th TNM system attributes AEG Siewert type II to esophageal classification system. However, the gastric and esophageal classification system which was more suitable for type II remains in disputation. This study aimed to illuminate the 8th TNM-EC or TNM-GC system which was more rational for type II, especially for patients underwent transhiatal approaches.

**Methods:**

We collected the database of patients with AEG who underwent radical surgical resection from two high-volume institutions in China: West China Hospital (*N* = 773) and Xi Jing Hospital of Fourth Military University (*N* = 637). The cases were randomly matched into 705 training cohort and 705 validation cohort. All the cases were reclassified by the 8th edition of TNM-EC and TNM-GC. The distribution of patients in each stage, the hazard ratio of each stage, and the separation of the survival were compared. Multivariate analysis was performed using the Cox proportional hazard model. Comparisons between the different staging systems for the prognostic prediction were performed with the rcorrp.cens package in Hmisc in R (version 3.4.4. http://www.R-project.org/). The validity of these two systems was evaluated by Akaike information criterion (AIC) and concordance index (C-index).

**Results:**

By univariate analysis, the HRs from stage IA/IB to stage IV/IVB were monotonously increased according to TNM-GC scheme in both cohorts (training 2.63, 3.91, 5.02, 8.64, 15.51 and 29.64; validation 1.54, 3.55, 4.91, 7.14, 11.67, 18.71 and 48.32) whereas only a fluctuating increased tendency was found when staged by TNM-EC. After the multivariate analysis, TNM-GC (*P* < 0.001), TNM-EC (*P* = 0.001) in training cohort and TNM-GC (*P* < 0.001) TNM-EC (*P* < 0.001) in the validation cohort were both independent prognostic factors. The C-index value for the TNM-GC scheme was larger than that of TNM-EC system in both training (0.721 vs. 0.690, *P* < 0.001) and validation (0.721 vs. 0.696, *P* < 0.001) cohorts. After stratification analysis for Siewert type II, the C-index for TNM-GC scheme was still larger than that of TNM-EC in both training (0.724 vs. 0.694, *P* = 0.005) and validation (0.723 vs. 0.699, *P* < 0.001) cohorts.

**Conclusions:**

The 8th TNM-GC scheme is superior to TNM-EC in predicting the prognosis of AEG especially for type II among patients underwent transhiatal approaches.

## Introduction

Although the incidence trend decreased monotonically over the past decades, gastric cancer still accounts for terrible cancer-related deaths and remains an enormous health burden globally [1–3]. In recent decades, more and more population-based researches that had illustrated the incidence of the adenocarcinoma in esophagogastric junction (AEG) presented a distinctive increased trend [4–6]. A retrospective study from our institution had also indicated the proportion of AEG among surgical patients had increased from 22.3 to 35.7% during the past 25 years [7]. When contrasted with the distal gastric adenocarcinoma, AEG might have its own heterogeneity and issued in a relatively worse prognosis [8]. Due to the distinguishing anatomic location, the classification of AEG had been always in debate during the past decades. As we all know, a rigorous and coherent staging system for AEG was crucial for clinicians to pick the optimal subsequent treatment regime [9, 10]. An accurate classification system AEG could also help clinicians make more suitable follow-up tactics and predict potential prognosis for these patients [11].

Currently, the American Joint Committee on Cancer (AJCC) Tumor-Node-Metastasis (TNM) staging system was the most common system that applied to solid tumors including gastric and esophageal cancers [12]. Numerous editions of staging system had been successively updated to reflect the prognosis as veritably as possible [13]. In the Fall of 2016, AJCC released the 8th edition of TNM classification which included many important modifications especially the redefinition for AEG: cancers with EGJ invasion that have their epicenter within the proximal 2 cm of the EGJ (Siewert type I/II) are to be staged as TNM-EC. Cancers whose epicenter is more than 2 cm distal from the EGJ, even if the EGJ is involved, would be staged by TNM-GC [12]. Thus, it could be seen that AEG II was still ascribed to TNM-EC.

The past seven editions of AJCC staging system ascribed AEG to TNM-EC system [14]; however, some researches had indicated that the staging scheme of AEG should be according to TNM-GC [15, 16]. Furthermore, some other reports had manifested that neither of these two systems could factually reflect prognosis of AEG and a new staging system should be introduced to this entity [17, 18]. In addition, the database from China for the new edition was relatively rare, whether the 8th staging system could really represent the prognosis of Chinese patients was still in suspicion [19, 20]. Even a further definition for the classification of AEG was established in the 8th edition, these two schemes which could better describe the prognosis of AEG especially for Chinese population was still ambiguous. To sum up, the classification system for AEG was always in controversy, gastric or esophageal scheme, which was more suitable for the classification of AEG (especially for type II) remained in discussion.

To the best of our knowledge, there were no large-sample reports to compare the classification of AEG according to the 8th edition of TNM-GC and TNM-EC. In consideration of these issues, we established a predictive nomogram by retrospectively analyzing database from two high-volume institutions in China to compare which system could better describe the classification of AEG (especially for type II) between TNM-GC and TNM-EC.

## Materials and methods

### Study population

**Fig. 1:**
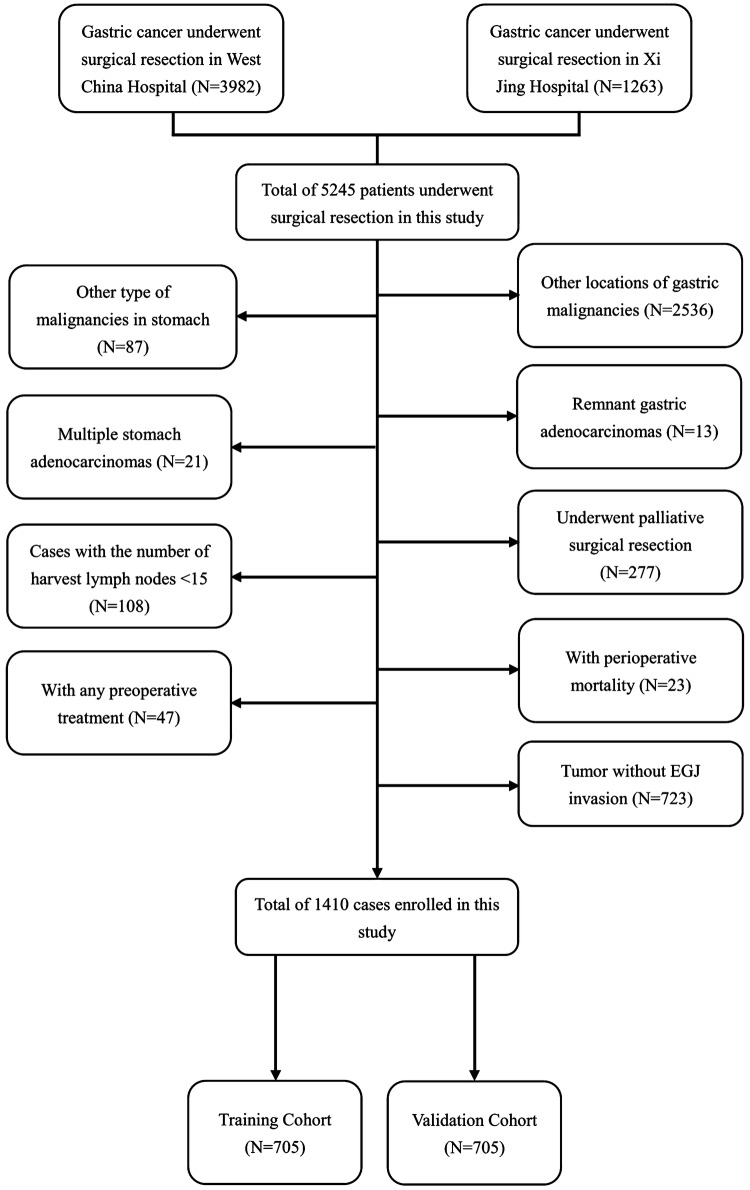
The flowchart of cases enrolled in this research

Total of 5245 consecutive patients with gastric cancer who underwent gastrectomy and distal esophagectomy in West China Hospital (WCH) from January 2002 to December 2013 and Xi Jing Hospital (XJH) from September 2006 to November 2013 were enrolled in this study. The diagnosis of primary AEG for all patients was confirmed by upper gastrointestinal endoscopy and biopsy before surgery. The inclusion criteria included (1) histologically confirmed gastric adenocarcinoma; (2) without any preoperative treatment; (3) tumor located in EGJ area with exact Siewert classification; (4) tumor with EGJ invasion; (5) with radical surgical resection (R0); (6) cases that the number of harvest lymph nodes ≥ 15; (7) the clinicopathological and the follow-up materials were completed. The exclusion criteria of our study include (1) other types of malignancies in stomach; (2) the middle and/or distal gastric adenocarcinoma; (3) the remnant gastric cancer; (4) with palliative surgical resection; (5) cases that had the number of harvest lymph nodes to be < 15; (6) tumors without EGJ invasion; (7) tumor with peritoneal dissemination or other distant organ metastasis; (8) received any preoperative treatment; (9) with perioperative mortality. The flow diagram is indicated in Fig. 1.

### Clinicopathological materials and surgical approaches for AEG

Clinicopathological data including demographic parameters, Siewert classification, tumor size (cm), the macroscopic type, pT–N–M stage, proportion of adjuvant therapy and degree of tumor differentiation (well, moderate, poorly differentiated and undifferentiated) were analyzed. The macroscopic type and pathological degree were reclassified according to Japanese gastric cancer treatment guidelines 2014 (ver. 4) [21]. Surgical-related parameters included resection pattern, number of lymph dissection, and combined organ resection. The major postoperative complications were defined as disease and/or disorder occurring due to gastrectomy that required reoperation or other interventions [22].

All enrolled patients in our study had obtained endoscopy, a computed tomographic scan for clinical tumor staging before surgery. The upper gastrointestinal barium meal was regularly conducted as auxiliary diagnosis for Siewert classification of AEG preoperatively, which also provided a reference for the surgical approach. For type II with distal esophagus invasion, transhiatal total gastrectomy (TG) or proximal gastrectomy (PG) combined with inferior mediastinal lymphadenectomy were preferred and type III tumors with transabdominal PG or TG in the department of gastrointestinal surgery from two institutions. The distance between EGJ and tumor epicenter was measured on the surgical specimens routinely. D2 was routinely performed, whereas PG plus D1/D1 + lymphadenectomy was selectively used for tumors in early stage. Intraoperative frozen section was a routine procedure to secure the tumor-free margins. For the reconstruction, Roux-en-Y anastomosis was mainly adopted for TG and esophagogastric anastomosis after PG. Combined organ resection was selectively performed to achieve a possible curative resection.

### New anatomical classification for AEG according to AJCC 8th edition

Siewert and Stein proposed a worldwide approved classification based on the anatomical position that divided AEG into three subgroups according to the distance from the epicenter to EGJ [23]. However, the 8th AJCC edition suggested that among tumor-invaded EGJ, only epicenter located within 2 cm upper and below the EGJ was designated as AEG [12]. In the view of the new edition, the traditional Siewert type II was authentic AEG, whereas the traditional type III was not part of AEG any more.

### TNM classification for AEG types II and III

In our research, TNM stage of AEG was recategorized in terms of the 8th AJCC cancer staging system for TNM-EC and TNM-GC, respectively [20]. However, the TNM-GC and TNM-EC had different definition for T3/4 stage, for instance, pT4a means tumor invaded serosa in TNM-GC [24], whereas pT4 means tumor invaded pleura, pericardium, azygos vein, diaphragm, or adjacent peritoneum in TNM-EC [12]. As we all know, unlike the gastric wall, the structure of esophageal wall has no serosa except the abdominal segment. Considering AEG was located between the abdominal esophagus and the stomach, pT4a staging of them was supposed to be attributed to TNM-GC scheme. The definition of N stage in terms of the number of metastatic regional lymph nodes and definition of M stage was consistent in both systems. Therefore, it was easy to see that the definitions of T, N, M categories were identical between TNM-EC and TNM-GC. The major difference between the two schemes consists in the stage subgroups.

## Follow-up and survival outcomes

In this study, overall survival (OS) was considered as the basic endpoint, which was calculated from date of surgical resection to the time of death from whatever cause or the latest follow-up (WCH: November 2017; XJH: May 2017). All the surgical patients were periodically followed up by outpatient visits, telephone interviews, network tools as well as letters. The follow-up interval was every 3–6 months during the first two postgastrectomy years, every 6–12 months during the subsequent 3 years, and all alive patients were followed annually thereafter until death [21]. Patients who were lost to the long-term follow-up were also recorded and the main causes of losses to follow-up were patients’ imparities to the outpatients’ visit and the alteration of contact information or address. Among the 5245 patients, 4782 (91.2%) had complete follow-up outcomes.

### Statistical analysis

The unordered categorical variables were assessed by Chi-square test or Fisher’s exact test. Continuous variables were compared using the *t* test or Mann–Whitney *U* test for variables with an abnormal distribution. Overall survival (OS) was calculated by Kaplan–Meier method life-table. The training and validation cohorts were matched with a proportion of 1:1 by Microsoft Excel 2016. Cox’s proportional hazard regression model with conditional backward stepwise was conducted to perform univariate and multivariate survival analyses. All the above evaluations were carried out with the SPSS version 22.0. The log-rank test and survival curves were employed to determine the significance of survival subgroups through R (version3.4.4. http://www.Rproject.org/). Cox proportional hazard regression model was also used to calculate Akaike information criterion (AIC) and concordance index (C-index) for each staging scheme to estimate their discriminatory ability and veracity, respectively in R. Previous studies indicated that a smaller AIC value represented a preferable model for predicting outcomes [25], whereas a larger C-index demonstrated a more accurate prognostic prediction [26]. The Nomogram and calibration curves were described by the package of Regression Modeling Strategies (http://CRAN.R-project.org/package=rms) in R. Comparisons between the different staging schemes for the prognostic prediction were conducted with the package of Harrell Miscellaneous (http://CRAN.R-project.org/package=Hmisc). The two-tailed *P* value that less than 0.05 were considered statistically significant.

## Results

### Demographics of study patients

Total of 1410 cases that fulfilled AEG II/III were admitted into our research for final analysis. There were 773 patients enrolled from West China Hospital and 637 patients from Xi Jing Hospital. The training and validation cohorts were rematched with a proportion of 1:1 in our study. The clinicopathological characteristics of these patients are indicated in Table 1. There was no significant difference in clinicopathological features between training and validation cohorts in this study.

**Table 1:** Clinicopathological and surgical-related parameters of patients with AEG types II and III

Demographic or characteristics	Training cohort (*N* = 705)	Validation cohort (*N* = 705)	*P* value
	No. of patients (%)	No. of patients (%)	
Gender (male/female)	580/125	576/129	0.782
Age (mean ± SD)	60.9 ± 9.4	60.3 ± 9.6	0.250
Siewert type (type II/III)	436/269	413/292	0.211
Maximal tumor size (cm)	5.2 ± 2.2	5.1 ± 2.3	0.352
Macroscopic type			0.191
Early gastric cancer	52 (7.4%)	45 (6.4%)	
Borrmannn-1	26 (3.7%)	25 (3.5%)	
Borrmannn-2	388 (55.0%)	357 (50.6%)	
Borrmannn-3	239 (33.9%)	278 (39.4%)	
Surgical approach			0.275
Total gastrectomy (TG)	558 (79.1%)	551 (78.2%)	
Proximal gastrectomy (PG)	147 (20.9%)	154 (21.8%)	
Extent of LN dissection			0.759
D1/D1+	97 (13.8%)	101 (14.3%)	
D2/D2+	608 (86.2%)	604 (85.7%)	
Combined organ resection	59 (8.4%)	62 (8.8%)	0.775
Adjuvant therapy			0.457
Yes	539 (76.5%)	526 (74.6%)	
No	166 (23.5%)	179 (25.4%)	
Histological grade			0.356
Well differentiated (G1)	42 (6.0%)	55 (7.8%)	
Moderately differentiated (G2)	233 (33.0%)	236 (33.5%)	
Poorly differentiated (G3)	430 (61.0%)	414 (58.7%)	
Mean number of harvested LNs	26.5 ± 12.4	26.1 ± 8.6	0.414
TNM-GC staging system^a^			0.218
IA	47 (6.7%)	44 (6.2%)	
IB	46 (6.5%)	57 (8.1%)	
IIA	89 (12.6%)	77 (10.9%)	
IIB	135 (19.1%)	112 (15.9%)	
IIIA	184 (26.1%)	189 (26.8%)	
IIIB	104 (14.8%)	137 (19.4%)	
IIIC	61 (8.7%)	54 (7.7%)	
IV	39 (5.5%)	35 (5.0%)	
TNM-EC staging system^b^			0.472
IA	8 (1.1%)	8 (1.1%)	
IB	25 (3.5%)	27 (3.8%)	
IC	32 (4.5%)	39 (5.5%)	
IIA	24 (3.4%)	21 (3.0%)	
IIB	66 (9.4%)	63 (8.9%)	
IIIA	27 (3.8%)	20 (2.8%)	
IIIB	243 (34.5%)	211 (29.9%)	
IVA	241 (34.2%)	281 (39.9%)	
IVB	39 (5.5%)	35 (5.0%)	
Postoperative complications	135 (19.1%)	138 (19.6%)	0.840

^a^AJCC 8th TNM staging system of gastric cancer

^b^AJCC 8th TNM staging system of esophageal cancer

For 773 cases in our institution, the proportion of Barrett’s esophagus was 6.0% (46/773). The distribution of histological degree among AEG combined with Barrett’s was: 13.0% with G1, 47.8% with G2 and 39.1% with G3 which indicated a higher rate of G1(5.0%) and G2 (29.0%) and lower rate of G3 (56.7%) when compared with patients without Barrett’s esophagus (*P* = 0.005).

### Hazard ratio for different TNM systems in two cohorts

**Fig. 2:**
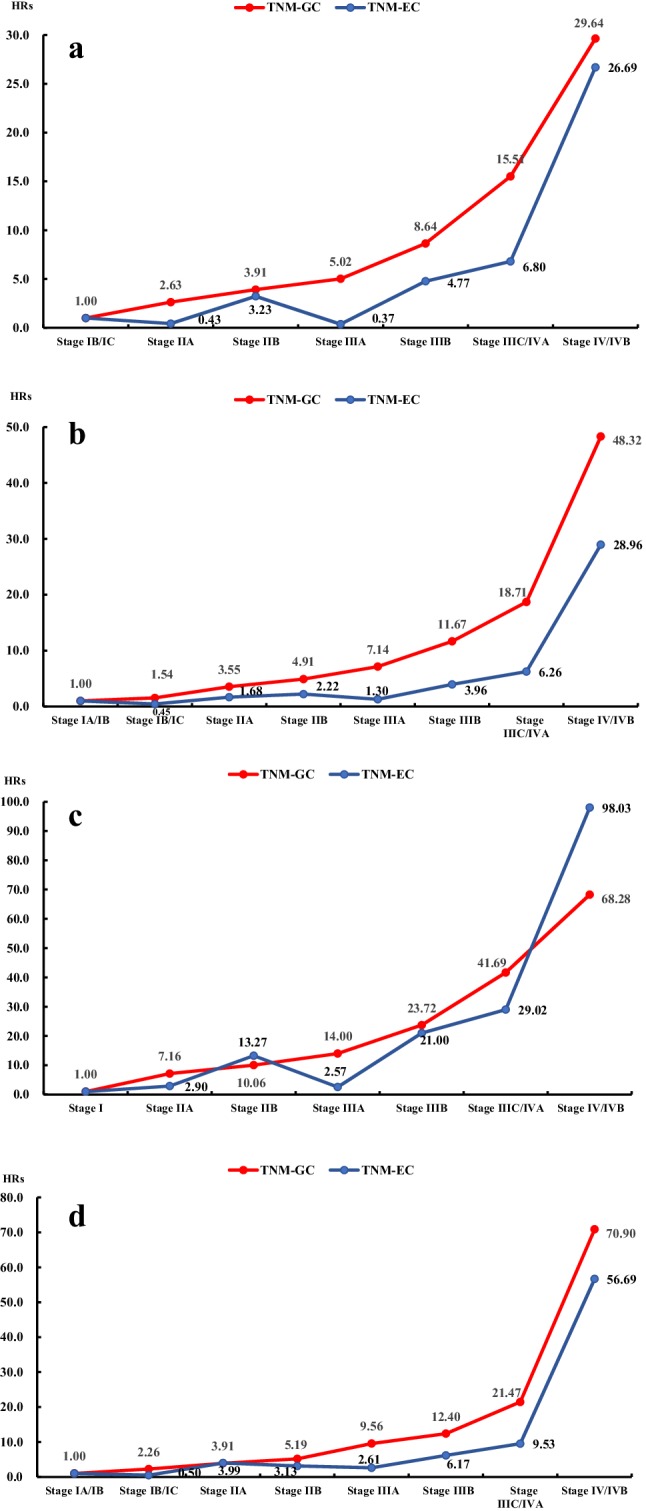
Hazard ratio of each stage subgroup to stage IA or IB according to the 8th AJCC classification: **a** AEG in training cohort; **b** AEG in validation cohort; **c** Siewert type II in training cohort; **d** Siewert type II in validation cohort

When compared with stage IA in TNM-GC and stage IA/IB in TNM-EC, the change of HRs from stage IA to stage IV in training and validation cohorts is indicated in Fig. 2a, b, respectively. The point estimate of the HRs between IA/IB/IC and IV/IVB increased in a stepwise manner with the stage subgroups in TNM-GC in both cohorts (training cohort: 1, 2.63, 3.91, 5.02, 8.64, 15.51, 29.64; validation cohort: 1, 1.54, 3.55, 4.91, 7.14, 11.67, 18.71, 48.32). However, in TNM-EC system, HRs only demonstrate a fluctuating increased trend from stage IA/IB to stage IVB in both cohorts (training cohort: 1, 0.43, 3.23, 0.37, 4.77, 6.80 and 26.69; validation cohort: 1, 0.45, 1.68, 2.22, 1.30, 3.96, 6.26, 28.96).

### The separation of survival among different subgroups

**Fig. 3:**
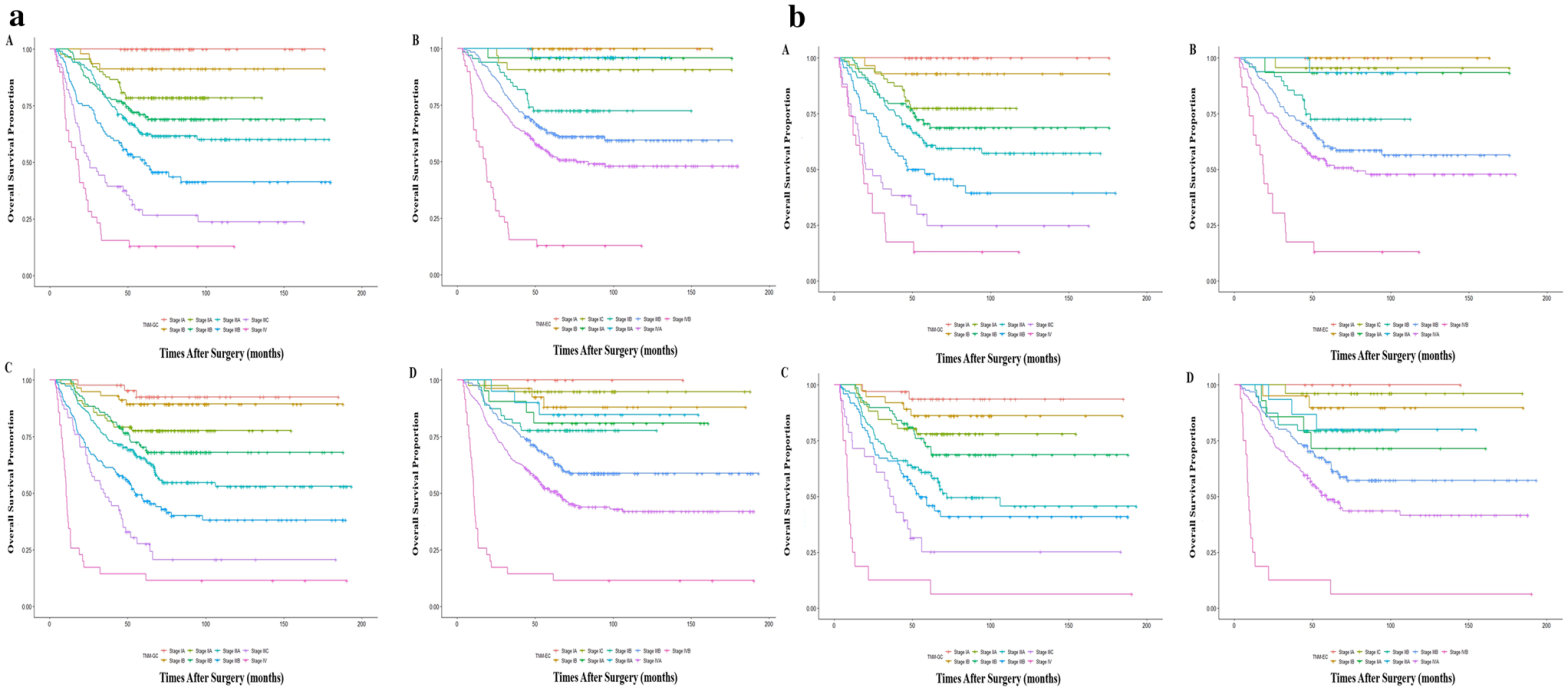
**a** Kaplan–Meier survival curves of total AEG (A. according to TNM-GC system in training cohort; B. according to TNM-EC system in training cohort; C. according to TNM-GC system in validation cohort; D. according to TNM-EC system in validation cohort); **b** Kaplan–Meier survival curves of AEG type II (A. according to TNM-GC system in training cohort; B. according to TNM-EC system in training cohort; C. according to TNM-GC system in validation cohort; D. according to TNM-EC system in validation cohort)

The survival curves in different stages were compared between close-by subgroups in terms of TNM-GC and TNM-EC in both cohorts. The average number of harvested lymph nodes in stage T1 was 24.9 ± 7.2 and 22.3 ± 9.2 in training and validation cohorts (*P* = 0.139), respectively. The survival difference between stages IA and IB, IIIA, IIIB, IIIC/IVA and IV/IVB was statistically significant in training cohort: TNM-GC (*P* = 0.040, *P* = 0.002, *P* = 0.006, *P* = 0.014) and stages IIA, IIB, IIIA, IIIB, IVA and IVB in TNM-EC system (*P* = 0.023, *P* = 0.011, *P* = 0.001, *P* = 0.010, *P* < 0.001), respectively. However, the significant survival difference only indicated between stages IIIB, IIIC/IVA and IV/IVB in validation cohort: TNM-GC (*P* = 0.002, *P* = 0.013, *P* < 0.001) and TNM-EC (*P* = 0.042, *P* = 0.001, *P* < 0.001) respectively. The 5-year survival rates illustrated a monotonous decreasing trend from stage IA to IV/IVB in both cohorts when according to the TNM-GC whereas TNM-EC only demonstrated a fluctuating decreased trend in both cohorts (Fig. 3a).

### Univariate and multivariate analyses of factors associated with prognosis

Table 2 indicates that tumor size, surgical approach, histological grade, TNM-GC and TNM-EC have significant correlation with prognosis in both training and validation cohorts. After Cox multivariate analysis, only TNM-GC (training: *P* < 0.001) (validation: *P* < 0.001) and TNM-EC (training: *P* = 0.001) (validation: *P* < 0.001) were independent prognostic factors in training and validation cohort, respectively.

**Table 2:** Univariable and multivariable analyses according to overall survival in two cohorts

Factors	Training cohort (*n* = 705)				Validation cohort (*n* = 705)			
	Univariable analysis		Multivariable analysis		Univariable analysis		Multivariable analysis	
	HR (95% CI)	*P* value	HR (95% CI)	*P* value	HR (95% CI)	*P* value	HR (95% CI)	*P* value
Gender (male/female)	0.799 (0.572–1.114)	0.186			0.869 (0.639–1.182)	0.372		
Age at surgery	1.057 (0.828–1.348)	0.658			0.986 (0.780–1.245)	0.904		
< 60								
≥ 60								
Siewert type (type II/III)	1.034 (0.807–1.325)	0.789			1.082 (0.856–1.367)	0.511		
Maximal tumor size (cm)		< 0.001*		0.772		< 0.001*		0.449
≤ 2								
2–5	2.576 (1.202–5.520)	0.015*	0.783 (0.362–1.692)	0.533	1.986 (1.163–3.392)	0.012*	0.692 (0.390–1.229)	0.209
5–10	4.172 (1.948–8.993)	< 0.001*	0.884 (0.405–1.932)	0.758	2.958 (1.735–5.045)	< 0.001*	0.781 (0.433–1.407)	0.666
> 10	7.176 (2.863–17.99)	< 0.001*	0.812 (0.311–2.116)	0.699	5.861 (2.689–12.78)	< 0.001^*^	0.960 (0.412–2.241)	0.925
Combined organ resection	1.260 (0.846–1.876)	0.255			1.128 (0.763–1.666)	0.546		
Macroscopic type	1.206 (0.933–1.560)	0.153			0.888 (0.696–1.133)	0.339		
Borrmann 0–1								
Borrmann 2–3								
Surgical approach (TG/PG)	1.601 (1.151–2.226)	0.005*	0.990 (0.700–1.398)	0.953	1.835 (1.345–2.504)	< 0.001*	1.282 (0.928–1.771)	0.132
Histological grade (G1–2/G3)	1.359 (1.053–1.755)	0.018*	1.016 (0.778–1.326)	0.910	1.410 (1.106–1.798)	0.006*	0.873 (0.673–1.133)	0.308
TNM-GC		< 0.001*		< 0.001*		< 0.001*		< 0.001*
TNM-EC		< 0.001*		0.001*		< 0.001*		< 0.001*

*TNM-GC* AJCC 8th gastric cancer staging system, *TNM-EC* AJCC 8th esophageal cancer staging system, *HR* hazard ratio, *95% CI* 95% confidence interval

*P < 0.05, statistical significance

### Comparison of predictive accuracy between TNM-GC and TNM-EC staging systems

As is shown in Table 3, the AIC and C-index values for both staging schemes were applied to further indicate the prognostic discriminatory and predictive veracity. When compared with TNM-EC, TNM-GC demonstrated a smaller AIC (3174.5 vs. 3231.7), (3410.4 vs. 3458.2) and larger C-index (0.721 vs. 0.690, *P* < 0.001) (0.721 vs. 0.696, *P* < 0.001) in both training and the validation cohorts.

**Table 3:** Comparison of predictive accuracy between the 8th TNM-GC and TNM-EC staging systems for AEG and type II patients

Training cohort (*N* = 705)	Concordance indices			
	*C* index	Bootstrap 95% CI	AIC	*P* value
TNM-GC system	0.721	0.691–0.751	3174.5	< 0.001*
TNM-EC system	0.690	0.659–0.721	3231.7	
Validation cohort (*N* = 705)				
TNM-GC system	0.721	0.692–0.750	3410.4	< 0.001*
TNM-EC system	0.696	0.665–0.726	3458.2	
Training cohort (*N* = 436) for Siewert type II				
TNM-GC system	0.724	0.686–0.762	1811.1	0.005*
TNM-EC system	0.694	0.655–0.733	1840.8	
Validation cohort (*N* = 413) for Siewert type II				
TNM-GC system	0.723	0.684–0.762	1756.8	< 0.001*
TNM-EC system	0.699	0.659–0.739	1785.2	

*TNM-GC* AJCC 8th gastric cancer staging system, *TNM-EC* AJCC 8th esophageal cancer staging system, *HR* hazard ratio, *95% CI* 95% confidence interval, *AIC* Akaike Information Criterion

*P < 0.05, statistical significance

**Fig. 4:**
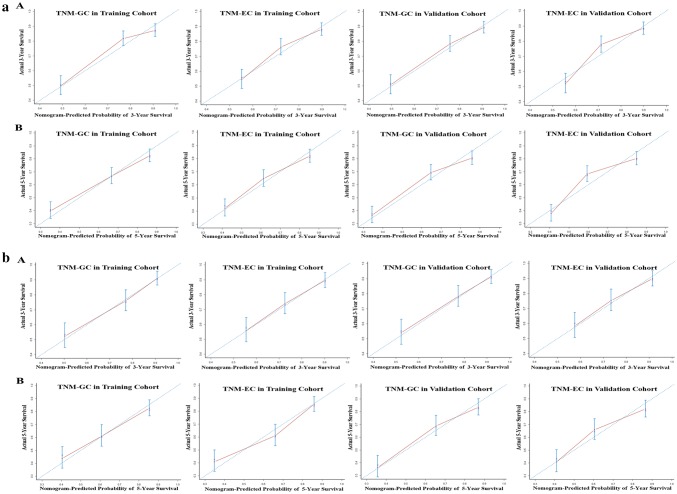
**a** The calibration curve for predicting all patients’ survival at 3 years (A) and 5 years (B) in both cohorts. **b** The calibration curve for predicting Siewert type II patients^,^ survival at 3 years (A) and 5 years (B) in both cohorts (Nomogram-predicted probability of overall survival is plotted on the *x*-axis; actual overall survival is plotted on the *y*-axis)

Nomograms based on TNM-GC and TNM-EC in both training and validation cohorts were employed to predict 3-year and 5-year survival. The calibration curves in two cohorts illustrated that TNM-GC predictive probability of 5-year survival was more approximate with the actual 5-year survival than that of TNM-EC (Fig. 4a). The TNM-GC system indicated a significant superiority over TNM-EC for predicting the prognosis of AEG.

### The outcomes of stratified analysis for Siewert type II

For Siewert type II, change of HRs from stage IA to stage IV in training and validation cohorts is indicated in Fig. 2c, d, respectively. When compared with stage I, a stepwise increased trend was observed in TNM-GC (training cohort: 1, 7.16, 10.06, 14.0, 23.72, 41.69, 68.28; validation cohort: 1, 2.26, 3.91, 5.19, 9.56, 12.40, 21.47, 70.90) and only a fluctuating increased trend was found in TNM-EC (training cohort:1, 2.90, 13.27, 2.57, 21.0, 29.02, 98.03; validation cohort: 1, 0.5, 3.99, 3.13, 2.61, 6.17, 9.53, 56.69) in both cohorts. Figure 3b reveals the survival curves of different stages of Siewert type II in both cohorts. The separation of the survival curves was better in TNM-GC staging system in both training and validation cohorts; however, a stepwise decreased trend of the 5-year survival rate was observed in TNM-GC in both cohorts, whereas only a fluctuating decrease trend was indicated in TNM-EC. Among AEG type II patients, when compared with TNM-EC, TNM-GC illustrated a smaller AIC (1811.1 vs. 1840.8) (1756.8 vs. 1785.2) and larger C-index (0.724 vs. 0.694, *P* = 0.005) (0.723 vs. 0.699, *P* < 0.001) in training and validation cohorts, respectively (Table 3). The nomogram of type II based on these two staging systems and the corresponding calibration curves in both cohorts also illustrated that the predictive probability of 5-year survival of TNM-GC was more close to the actual 5-year survival than that of TNM-EC (Fig. 4b).

## Discussion

Recently, there were many reports that concern about the increased trend of AEG and indicated that its unique features differ from common gastric and esophageal adenocarcinomas [4–6, 27]. A veracious staging system for AEG was the primary basis for evaluating prognosis, making suitable strategy on stage-specific treatments [17]. However, there were also many different opinions on the classification of AEG that still left the propensity of the staging system of AEG in ambiguity [15–17, 28]. The 8th edition made the definition of AEG more clear and still ascribed AEG II to TNM-EC [12, 19, 29]. However, whether the definition was indeed adequate for AEG II especially for Chinese population was still unknown. Our study demonstrated that TNM-GC had a significant advantage over TNM-EC with regard to the correlation between HR and stage, the separation of the survival among different stages in both training and validation cohorts. The effectiveness and accuracy of TNM-GC were better than TNM-EC; the stratified analysis also indicated the consistent results for Siewert type II in both cohorts. These results might have an influence on subsequent TNM staging system revision for AEG.

A monodirectional increased trend of HRs was observed in TNM-GC in both training and validation cohorts; however, when classified by TNM-EC, only the fluctuating increase was found between stages IIB and IIIB. These might indicate that TNM-EC was insufficient in describing the survival trend in these stages. One possible explanation was that patients in stage IA and IB were too rare to produce a credible value of HRs in these three subgroups when in terms of TNM-EC. On the other hand, the survival has no significance from stage IA to II A in TNM-EC in both cohorts. The similar outcome was also observed from a study in Japan [15]. An adverse survival curve was detected in stages IC, IIA, IIB and IIIA in both cohorts when according to TNM-EC. The stratification analysis for AEG II also indicated similar outcomes. All these above results might demonstrate that the discrimination and factuality of TNM-EC were insufficient for AEG type II. The following comparison between two Cox multivariable models further ascertains that TNM-GC plays a more important role in predicting the prognosis for AEG type II in both cohorts. The prognostic model which included TNM-GC might more efficient than that included TNM-EC, a smaller AIC and larger C-index were dug out in both training and validation cohorts and consistent outcomes were detected in AEG II in both cohorts.

As many previous reports, the different surgical approaches combined with different lymph node dissection could bring different survival outcomes. Resection of more lymph nodes could bring to a better prognosis and a more accurate survival estimation [30, 31]. Recent researches further indicated that the extent of lymphadenectomy is intimately related to the overall survival of patients with AEG [32]. In our study, when compared with transthoracic approach, a deficient upper mediastinal lymphadenectomy may occur via transhiatal approach. The scanty number of harvest lymph nodes might also cause stage migration. On the other side, the lymph node metastatic manner and tumorigenesis in this region may be different from genuine esophageal adenocarcinoma [33]. These may partly paraphrase the discrimination of survival outcomes from esophageal adenocarcinoma. For AEG II patients, the issue of surgical approach (transthoracic or transhiatal) and lymphadenectomy had been always in debate [31, 34, 35]. Different surgical approaches were performed in different departments and combined with various lymphadenectomies. These could result in different survival outcomes for AEG II patients [31, 34]. Considering the potential bias brought by surgical approaches, we exclude AEG II patients who underwent transthoracic resection. In our study, the outcomes could indicate that TNM-GC was superior over TNM-EC for AEG II patients who underwent transhiatal approach with sufficient abdominal lymph node dissection.

In line with some former reports [16, 36], the classification of AEG type II should be considered as a part of gastric cancer. Our study also illustrated that TNM-GC manifested a superior heterogeneity compared with TNM-EC. The anatomical and adjoining structures of type II were more semblable to stomach instead of esophagus and the structure of gastric wall in the position of type II was same with stomach [37]. On the other hand, the location of type II that squamous epithelium transformed to glandular epithelium is rather different from the squamous epithelium of esophagus. Different epithelial ingredients with different tumorigenesis might induce discrepant prognosis. However, the pathological components in type II have become glandular epithelium which was more similar to typical gastric epithelium [37]. The tumorigenicity in this position might have something in common with typical gastric adenocarcinoma. A recent basic research also indicated that the esophageal adenocarcinomas had strong similarity with the chromosomally unstable variant of gastric adenocarcinoma, suggesting that these cancers including AEG were more similar with gastric adenocarcinoma in molecular level or should be considered as a single disease entity [38]. In summary, Siewert type II might have more common with gastric adenocarcinoma from the point of anatomy and pathology.

The former research had found more proportion of undifferentiated type in gastric cardia cancer leads to worse prognosis [39]. The histological grade was required for the final stage in the TNM-EC in the 8th edition. However, Kim et al. reported that histological factor might recede the prediction of patient survival for AEG [40]. The groups with G1/2 differentiated adenocarcinoma indicated better survival than those with G3 in training cohort. Our report indicated that histological grade had a significant correlation with prognosis in univariate analysis in both training (HR = 1.359) and validation (HR = 1.410) cohorts; however, histological grade was not an independent prognostic factor for AEG after multivariable analysis in both cohorts. Consequently, whether histologic grade should be applied to the final staging system of AEG is required to be prudently considered further.

In summary, many factors may have different impacts on the prognosis of AEG, the existent 8th TNM staging system might still have some insufficiency in predicting the prognosis. Therefore, a veracious staging system that is suitable for AEG accurately needs to have further consideration [32]. As mentioned in our research, both schemes were defective in representing the survival of stage IA–IIA. One reason was the relatively low proportion of early staging cancer in China and the number of patients in these stages was too rare to reflect the distinctiveness. An initiate staging system specific for AEG was also demanded to describe the prognosis exactly in the future.

This research has several limitations. In the first place, there was no Siewert type I cancer enrolled. In the second, we did not include AEG II patients who underwent transthoracic approach, all the patients in our study were only treated by transhiatal gastrectomy and not received upper mediastinum lymphadenectomy. Third, the number of patients in stages I and II was relatively small to reflect the discriminative prognosis of AEG and the stage relatively concentrated to stage III in our study which may also have an impact on the overall survival outcomes.

## Conclusions

In conclusion, the staging system of AEG types II and III should be according to TNM-GC system when compared with TNM-EC systems. TNM-GC system is superior to TNM-EC in predicting the prognosis of AEG type II.
